# Clinical and Therapeutic Challenges in Aggressive Diffuse Large B-Cell Lymphoma of Immune-Privileged Sites: A Case Report of Orbital and Cervical Involvement in an End-Stage Renal Disease (ESRD) Patient

**DOI:** 10.7759/cureus.90154

**Published:** 2025-08-15

**Authors:** Ravada R Hemanth Sai Sri Harsha, Sandeep Garg, Praveen Bharti, Mahima Mehra, Manidipa Mondal

**Affiliations:** 1 Medicine, Maulana Azad Medical College, New Delhi, IND; 2 Medicine, Lok Nayak Hospital, New Delhi, IND

**Keywords:** cervical lymphadenopathy, extranodal involvement, immune-privileged sites, non-hodgkin lymphoma, optic nerve, orbital lymphoma, paraspinal muscle infiltration, retrobulbar mass

## Abstract

Diffuse large B-cell lymphoma (DLBCL) is an aggressive (fast-growing) non-Hodgkin lymphoma (NHL) that affects B-lymphocytes. We report a rare and diagnostically complex case of aggressive DLBCL manifesting in immune-privileged and atypical extranodal sites of the head and neck, including bilateral retrobulbar regions, optic nerves, zygomatic muscle, cervical soft tissues, and paraspinal musculature, in a 70-year-old male patient with multiple comorbidities including end-stage renal disease (ESRD) and prior cerebrovascular accident. The patient presented with rapidly progressive bilateral proptosis and systemic signs of sepsis. Diagnostic imaging, constrained by renal insufficiency, revealed multifocal soft tissue masses without intracranial involvement. Histopathology and immunohistochemistry confirmed nongerminal center subtype DLBCL (CD20+, BCL2+, MUM1+, MYC-, CD3-, CD7-). Bone marrow analysis excluded systemic leukemic-phase disease. Due to the patient's frailty and organ dysfunction, standard rituximab, cyclophosphamide, doxorubicin, vincristine, and prednisone (R-CHOP) therapy was contraindicated; a modified immunochemotherapy with corticosteroids and rituximab was initiated, yielding a partial reduction in tumor size. Despite initial improvement, the patient succumbed to complications of multiorgan failure. This case highlights the clinical and therapeutic challenges in diagnosing and managing extranodal DLBCL in elderly, immunocompromised patients, especially when the disease involves immune-privileged sites and standard diagnostic tools are limited.

## Introduction

Diffuse large B-cell lymphoma (DLBCL) is an aggressive (fast-growing) non-Hodgkin lymphoma (NHL) that affects B-lymphocytes. Primary DLBCL is one of the most aggressive subtypes of NHL, predominantly affecting the elderly population. However, its occurrence in immune-privileged regions of the head and neck is exceedingly rare [1]. DLBCL accounts for approximately 30% of all NHL cases, with the majority originating from nodal tissues; nonetheless, about 30% of patients present with extranodal involvement at diagnosis [2]. The most commonly affected extranodal sites include the gastrointestinal (GI) tract, skin and soft tissues, skeletal system, and the urogenital tract. As the disease progresses, it may disseminate to the bone marrow, pleura, peritoneum, and central nervous system (CNS), often obscuring the identification of the original site of onset. Immune-privileged sites refer to anatomical locations where local immune surveillance is reduced, allowing tumor cells to survive and sometimes relapse despite systemic control. These are very uncommon sites when compared to nodal/GI involvement of DLBCL. They are also usually associated with aggressive histology and higher relapse risk. The CNS, eyes, and testes are recognized as immune-privileged sites, wherein lymphocytic cells can evade immune surveillance, thereby receiving reduced immunologic and chemotherapeutic clearance. This unique immunological environment contributes to both the pathogenesis and the therapeutic challenges associated with lymphomas arising in these regions, underscoring their clinical complexity and resistance to standard treatment modalities [3]. CNS involvement occurs in approximately 10% of patients with NHL, with optic nerve infiltration reported in about 5% of these cases. The frequency of CNS involvement varies depending on the histological subtype and the aggressiveness of the lymphoma. Notably, as therapeutic advancements have extended patient survival and reduced mortality rates, the incidence of CNS manifestations appears to be increasing. Consequently, ophthalmologists are more likely to encounter patients presenting with ocular complications as sequelae of NHL [4]. Metastatic involvement of the eye and orbit most commonly affects the choroid, likely due to its rich vascular supply. According to a clinicopathologic review conducted by the Armed Forces Institute of Pathology, isolated optic nerve metastasis accounts for approximately 1.3% to 12% of all ocular and orbital metastases [5]. The most frequent primary sources of optic nerve metastases are breast carcinoma (25-33%) and lung carcinoma (11-15%), mirroring the distribution of the most prevalent primary tumor metastasizing to the eye and orbit [6]. Intraorbital lymphoma was relatively common compared to optic nerve and retroorbital lymphomas. Only a few number of case reports have been recorded in the literature regarding large cell lymphomas arising from the optic nerve and the retroorbital area. We are reporting a rare case of DLBCL involving immune-privileged sites like the optic nerve and other head, neck areas, presenting initially with bilateral asymmetrical proptosis, later multiple swellings in the right temporal region and around the neck, along with multiple comorbidities that include end-stage renal disease (ESRD). We were also emphasizing on difficulties that occur in staging and drug therapy of DLBCL in the setting of ESRD due to difficulties in administering contrast agents, complications that interfere with the dosing of the drug, availability of hemodialysis, and increased risk of adverse reactions to chemotherapy drugs.

## Case presentation

We report the case of a 70-year-old male patient with a long-standing history of hypertension and hypothyroidism, managed over the past two decades with regular use of amlodipine, atenolol, and thyroxine supplementation. He was a retired shopkeeper, living with his 68-year-old spouse and six children. The patient had a remote history of occasional smoking (1-2 cigarettes per day) for approximately 20 years, which he discontinued upon his initial diagnosis of hypertension and hypothyroidism.

**Figure 1:**
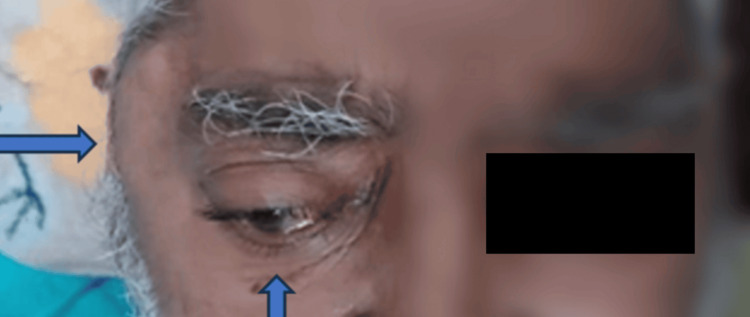
Picture of the patient having asymmetrical proptosis, also had swelling over the right zygoma (Pictograph taken with the permission of family members)

One year prior to presentation, he experienced a cerebrovascular accident resulting in significant functional decline. Since then, he had been predominantly bedridden and unable to independently perform activities of daily living. Six months prior to admission, he was diagnosed with chronic kidney disease (CKD) following persistently abnormal renal function test results; however, he was managed conservatively without initiation of dialysis. Two months before admission, he developed progressive right-sided proptosis (Figure 1) followed by similar involvement of the left eye. 

Over the subsequent month, multiple swellings appeared over the right zygomatic region (Figure 1) and the posterior aspect of the neck. Additional swellings over the anterior neck were noted approximately 20 days before hospital admission. He presented to the Department of Medicine at Lok Nayak Hospital with clinical features consistent with complications of ESRD. His presenting symptoms included bilateral pedal edema, abdominal distension, dyspnea, and altered sensorium for one week. His family also reported a cough with minimal yellowish sputum production. On initial examination, the patient was hypotensive and tachycardic and had relatively warm extremities. He was brought in with altered mentation and a poor Glasgow Coma Scale (GCS) score. Chest auscultation revealed coarse crepitations bilaterally, while abdominal examination demonstrated ascites without evidence of hepatosplenomegaly. A comprehensive clinical evaluation was performed upon admission (Table 1), including routine laboratory investigations such as a complete hemogram, serum electrolytes, hepatic function tests, and renal function tests.

**Table 1:** Important biochemical and hematological investigations done WBC: white blood cell; A/G ratio: albumin/globulin ratio; IgG: immunoglobulin G

Test	Day 2	Day 5	Reference range
Hemoglobin (g/dL)	7.2	8.9	13.5-17.5 (males)
WBC count (/mm^3)^	24000	17000	4500-11,000
Neutrophils (%)	95	90	50-70
Platelets (cells/dL)	90000	130000	150000-450000
Blood urea (mg/dL)	174	136	7-20 (adults)
Serum creatinine (mg/dL)	10.6	9.1	0.3-1.4
Serum sodium (meq/L)	129	136	135-145
Serum potassium (meq/L)	6.2	5.9	3.4-4.5
Calcium (mg/dL)	12	10.1	8.4-10.6
Phosphate (mg/dL)	6.9	5.3	2.1-4.7
Procalcitonin (ng/ml)	43.2	24	<0.05
Antinuclear antibody	01:40	-	<1:40
Carcinoma embryonic antigen (ng/mL)	3.2	-	0-2.5
Serum IgG (mg/dL)	756.3	-	600-1700
A/G ratio	0.75	-	1.1-2.5
Total protein (g/L)	6.2	-	6.0-8.3

Additionally, a baseline chest radiograph and abdominal ultrasonography were conducted. Serial laboratory investigations revealed significant abnormalities indicative of severe systemic illness, renal impairment, and evolving sepsis.

On day 2, hemoglobin levels were decreased, consistent with anemia. A slight increase was noted by day 5, though levels remained below the expected range. The white blood cell count was raised initially and showed a decreasing trend over the observation period. Neutrophilic predominance persisted throughout, suggestive of a bacterial infection. Platelet counts remained decreased, without normalization during the course, indicating thrombocytopenia potentially associated with sepsis or bone marrow involvement. Renal function tests revealed markedly raised blood urea and serum creatinine levels on day 2, with a declining trend by day 5, though values remained well above the reference limits, consistent with significant renal impairment. Electrolyte analysis demonstrated an initial decrease in serum sodium followed by an upward trend, while potassium, calcium, and phosphate levels were raised initially and gradually decreased over time, reflecting metabolic disturbances associated with advanced renal dysfunction. Procalcitonin levels were markedly raised on day 2 and showed a decreasing pattern by day 5, suggestive of systemic bacterial infection. Immunological testing revealed a weakly positive antinuclear antibody at the upper limit of normal. Carcinoembryonic antigen levels were mildly raised, prompting further evaluation for potential neoplastic processes. Serum IgG remained within the normal range. The albumin/globulin ratio was decreased, indicating a possible chronic inflammatory state or hepatic dysfunction. Total protein was at the lower limit of normal.

**Figure 2:**
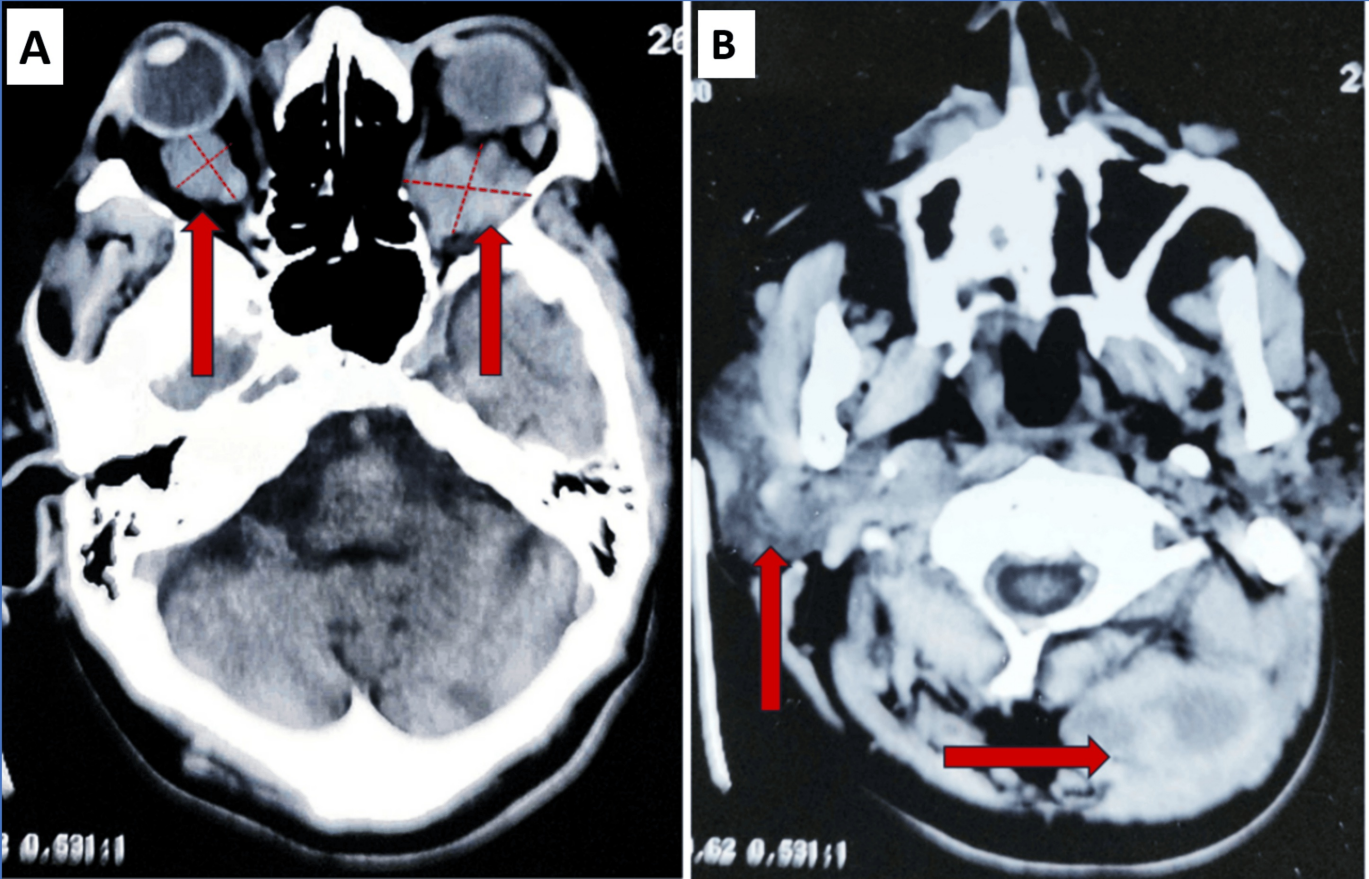
(A) Noncontrast CT head showing bilaterally asymmetric homogenous lobulated masses which is slightly hyperdense compared to extraocular muscles one on the right side and another retroorbital mass on the left side. (B) Some deposits of tumor in paraspinal muscles of neck evident as hypointense areas

Peripheral smear analysis demonstrated the presence of toxic granulations, and the red cell morphology was consistent with a normocytic, normochromic pattern. Arterial blood gas (ABG) analysis indicated severe metabolic acidosis accompanied by elevated lactate levels, suggestive of tissue hypoperfusion. These findings are consistent with a clinical picture of advanced renal dysfunction, systemic inflammatory response suggestive of sepsis, and potential underlying neoplastic etiology. Noncontrast CT and MRI have been planned to rule out the cause of proptosis and multiple swellings on his body. Primarily, we kept a differential diagnosis of the mass as secondary with occult primary malignancy. Given the patient's ESRD, noncontrast MRI and CT scans were employed to minimize the risk of contrast-induced nephropathy. Imaging revealed bilaterally asymmetric, homogeneous, lobulated orbital masses (Figure 2). The mass on the right side was slightly hyperdense relative to the extraocular muscles, while a retroorbital mass was noted on the left side. Additionally, there was evident enlargement and mild hyperdensity within the right zygomatic muscle, suggestive of tumor infiltration. Further findings included hypointense deposits within the paraspinal muscles of the neck on MRI, indicative of possible metastatic or infiltrative tumor involvement. The overall imaging profile raised a strong suspicion for a lymphoproliferative or metastatic process with multifocal soft tissue involvement, including orbital and paraspinal musculature.

**Figure 3:**
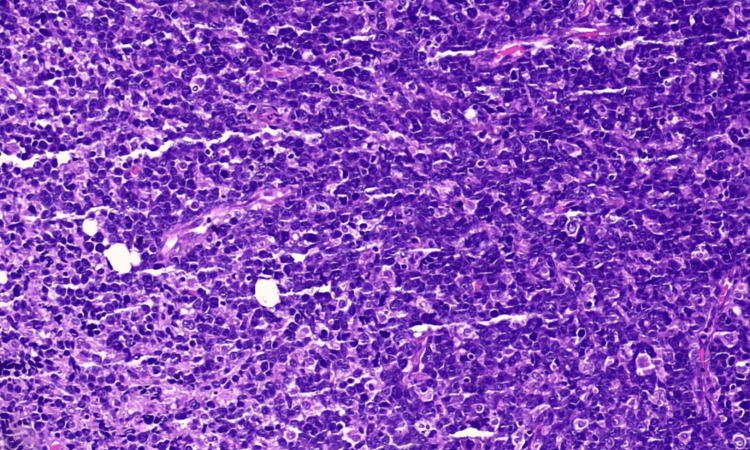
Histopathology and immunohistochemical staining of the specimen resected. Morphology of DLBCL, NOS with cells showing diffuse growth pattern with relatively few small lymphocytes with variable degree of mitosis and apoptosis nuclei with variable size and contour DLBCL, NOS: diffuse large B-cell lymphoma, not otherwise specified

**Figure 4:**
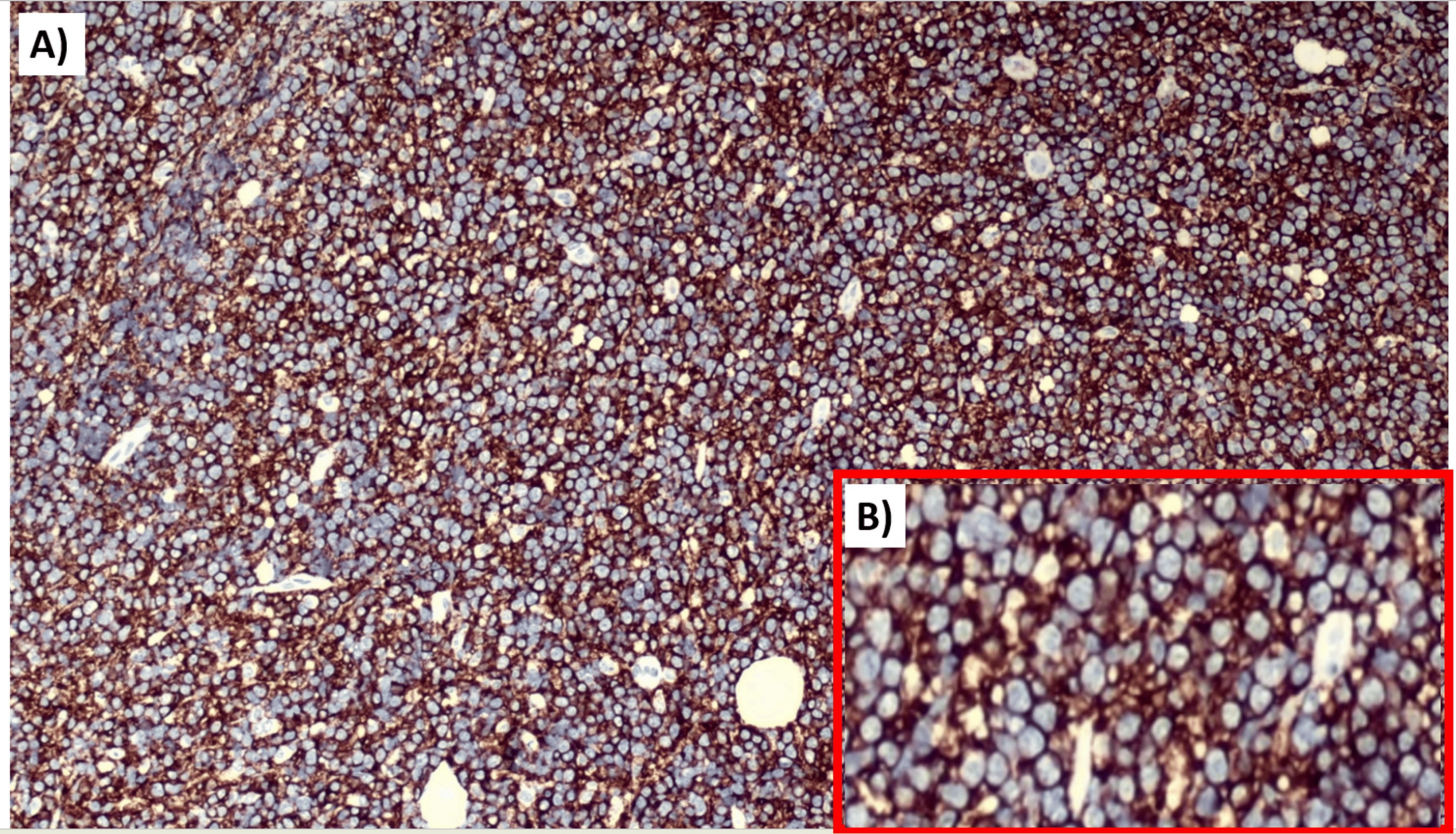
(A) Strongly positive cells for CD20. (B) Magnified image showing CD20 positivity

**Figure 5:**
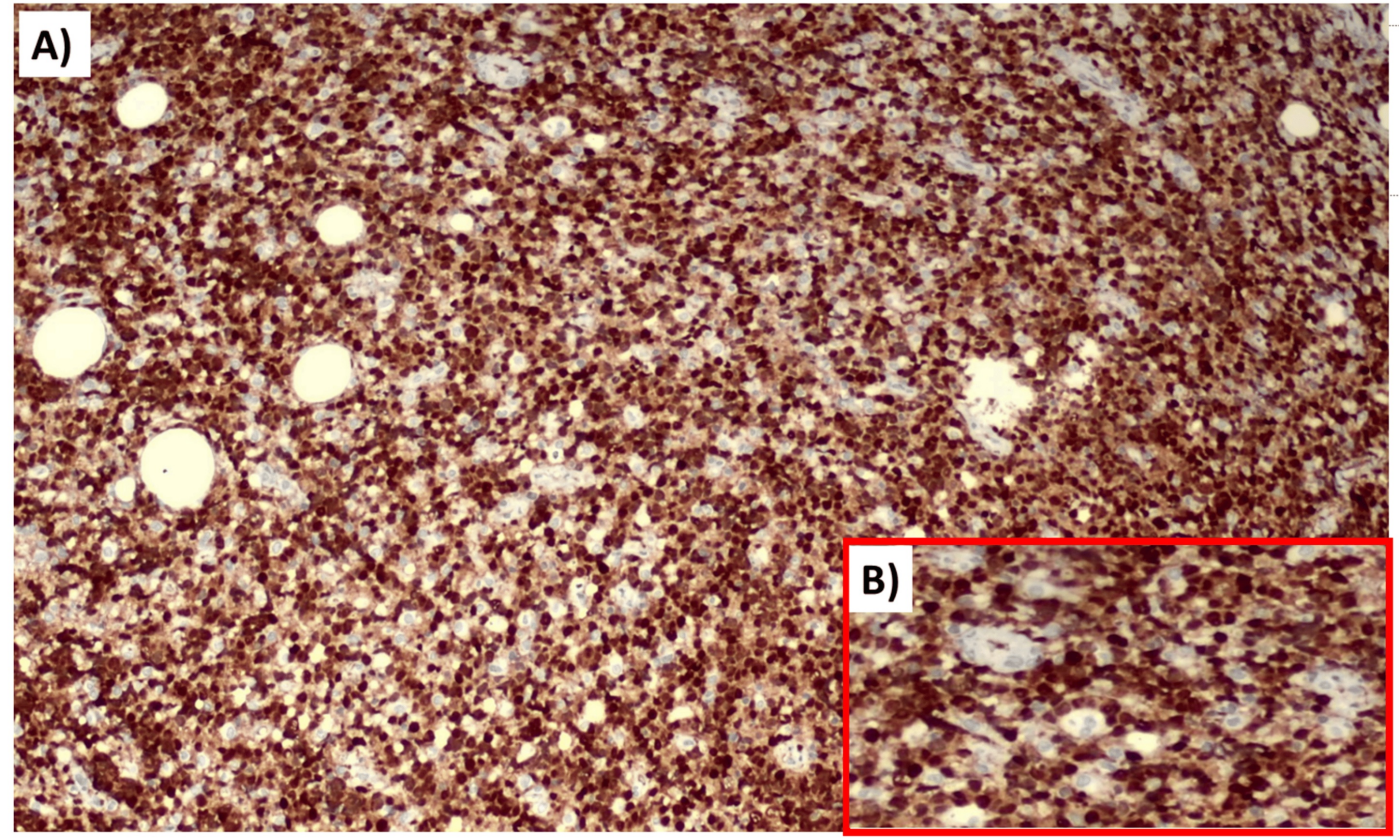
(A) Strongly positive cells for MUM1. (B) Magnified image showing cells with mum1 positivity

**Figure 6:**
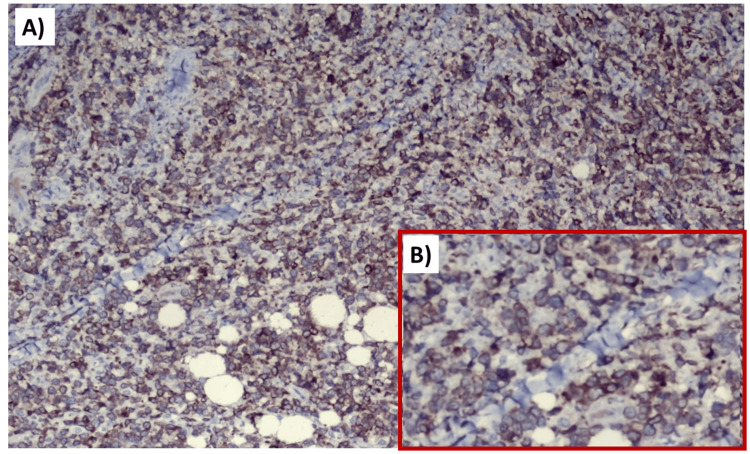
(A) Strongly positive cells for BCL2. (B) Magnified image showing cells with BCL2 positivity

Importantly, no evidence of intracranial masses was observed on imaging, effectively excluding a primary intracranial origin of the orbital lesions. To investigate potential GI primary tumors with secondary metastases, both upper and lower GI endoscopy were performed; neither revealed any luminal masses or suspicious lesions. Noncontrast imaging of the chest and abdomen was largely unremarkable, with the exception of multiple small peritoneal deposits. However, due to the absence of corresponding lymphadenopathy or visceral organ involvement, these findings were deemed of limited diagnostic significance. No abdominal lymph node enlargement was noted, although a few lymph nodes in the deep cervical fascia were found to be enlarged. Given the diagnostic uncertainty and the multifocal soft tissue involvement, histopathological and cytological analyses were pursued. Surgical excision of one of the orbital masses was performed, along with a biopsy of an enlarged cervical lymph node on the right side. Histopathological examination (Figure 3) of the orbital mass reveals the features consistent with DLBCL, not otherwise specified (NOS). The specimen demonstrated a diffuse growth pattern with relatively few small lymphocytes and variable degrees of mitosis and apoptosis. The neoplastic cells exhibited large nuclei, some measuring three to four times the size of normal lymphocytes, with irregular nuclear contours. Immunohistochemical staining was strongly positive for CD20 (Figure 4), BCL29 (Figure 5), and MUM1 (Figure 6), and negative for MYC, CD3, and CD7, supporting a diagnosis of nongerminal center subtype of DLBCL.

A final diagnosis of aggressive DLBCL with bilateral retrobulbar involvement and multifocal infiltration of atypical extranodal sites, including the left paraspinal neck muscles, right zygomatic region, and cervical soft tissues, was established. The patient presented emergently with uremic complications of ESRD and systemic sepsis, further complicating the clinical picture. To evaluate for potential marrow involvement given the aggressive dissemination, a bone marrow biopsy was performed (Table 2).

**Table 2:** Examination findings of the bone marrow

Parameter	Description
Erythroid series	Normoblastic reaction with few proerythroblasts
Myeloid series	Unremarkable, showing maturation up to the neutrophil stage
Megakaryocytes	Adequate, with a mild rise in lymphoid cells
Hemosiderin-laden macrophage	Few
Final impression	Cellular reactive marrow showing normal tri lineage hematopoiesis

The examination revealed normocellular marrow with no evidence of lymphomatous infiltration. Repeated peripheral blood smears showed no atypical cells, and flow cytometric analysis of peripheral blood was within normal limits, effectively ruling out leukemic-phase lymphoma or circulating lymphoma cells.

Given the patient's advanced age, frailty, and significantly compromised cardiac and renal reserves, a decision was made to initiate a modified and less intensive chemotherapy regimen, R-mini-CHOP. The proposed protocol included rituximab 375 mg/m², cyclophosphamide 400 mg/m², doxorubicin 25 mg/m², vincristine 1 mg (all administered on day 1 of each cycle), and prednisolone administered orally from day 1 to day 5. However, due to the patient's critical condition, marked by mechanical ventilation for respiratory support, poor cardiac reserve, ESRD, and active sepsis, only a limited therapeutic approach could be undertaken.

After achieving control of sepsis through appropriate supportive care and antimicrobial therapy, the patient was initiated on glucocorticoids alone, along with a single dose of rituximab. Remarkably, a partial clinical response was observed: there was a visible reduction in orbital proptosis and a decrease in the size of head and neck swellings, indicating chemosensitivity of the disease.

Despite these initial improvements, the patient’s overall clinical status continued to decline, and he ultimately succumbed to complications arising from multiple end-stage comorbidities, including renal failure, cardiac insufficiency, and sepsis.

## Discussion

We present a rare and diagnostically challenging case of aggressive DLBCL, a subtype of NHL, atypically involving multiple head and neck regions, including bilateral retrobulbar spaces, the right zygomatic region, cervical soft tissues, and paraspinal muscles. The complexity of this case was further heightened by the patient's ESRD and multiple comorbidities, including prior cerebrovascular accident and sepsis, which significantly limited the extent of diagnostic and therapeutic interventions. Diagnosis and staging of lymphoma in patients with ESRD pose a considerable challenge due to contraindications for contrast-enhanced imaging and limitations in performing a positron emission tomography (PET)-CT scan, the current gold standard for accurate staging [7]. Consequently, staging in this case relied on noncontrast imaging modalities and was necessarily imprecise. Despite these limitations, the patient was diagnosed with a disseminated and highly aggressive form of DLBCL based on histopathological and immunohistochemical evaluation. DLBCL is known for its aggressive behavior and frequent extranodal involvement, accounting for approximately 30% of all NHLs [8,9]. While orbital lymphomas are typically indolent and often marginal zone B-cell lymphomas, DLBCL of the orbit is rare and exhibits a locally invasive pattern, with potential extension into periorbital bones, paranasal sinuses, and, less commonly, the intracranial compartment [3]. The optic nerve and globe involvement is particularly uncommon, making this case unusual in its anatomical presentation. According to the World Health Organization (WHO) classification, DLBCL is further subdivided into cell-of-origin (COO) subtypes: germinal center B-cell-like (GCB) and activated B-cell-like (ABC) subtypes [10,11]. Approximately 10-15% of DLBCLs remain unclassifiable, and immunophenotyping and morphological analysis are critical to distinguish between B-cell and T-cell lymphomas, using markers such as CD3, CD5, CD20, and CD79a. Once a B-cell phenotype is confirmed, further immunohistochemical profiling, typically including BCL2, BCL6, CD10, CD23, CD30, Cyclin D1, MUM1, and light chains (κ, λ), is used for subtype identification and prognostication [12,13]. In our patient, immunohistochemistry demonstrated positivity for CD20, BCL2, and MUM1, with negative expression of CD3, CD7, MYC, BCL6, and CD10, consistent with a nongerminal center (non-GCB) subtype. Ki-67, a proliferation marker with prognostic value, is associated with a clinical course and immunophenotype that support a high-grade, rapidly progressive disease [8]. However, we found the Ki-67 marker to be negative. Our patient was also negative for MYC and BCL6 translocations, which are associated with very poor outcomes [12]. The non-GCB subtype is known to be more aggressive and less responsive to standard chemotherapy than its GCB counterpart. The standard first-line regimen for DLBCL is rituximab, cyclophosphamide, doxorubicin, vincristine, and prednisone (R-CHOP), which cures approximately 60% of patients [2]. However, for elderly or frail individuals with significant comorbidities, dose-adjusted regimens such as R-mini-CHOP are often considered to reduce treatment-related toxicity [14].

In this case, although R-mini-CHOP was initially planned, the patient's critical condition, including mechanical ventilation, hemodynamic instability, and poor renal function, necessitated a more conservative approach. He was started on glucocorticoids and a single dose of rituximab, to which he demonstrated a partial response, evidenced by a reduction in orbital proptosis and regression of soft tissue swellings. Nevertheless, despite initial improvement, the patient ultimately succumbed to complications of his underlying multiorgan failure, including ESRD and cardiac insufficiency. This case underscores the diagnostic and therapeutic complexities associated with extranodal, immunoprivileged sites, involving DLBCL in elderly patients with multiple comorbidities. It also highlights the need for individualized treatment strategies, particularly in resource-limited or medically challenging contexts where standard diagnostic and therapeutic modalities cannot be safely applied.

## Conclusions

This case highlights a rare and aggressive presentation of DLBCL with extensive involvement of atypical and immune-privileged extranodal sites, including bilateral retrobulbar regions, cervical musculature, and the zygomatic area, in the setting of ESRD and multiple comorbidities. The diagnostic process was complicated by limitations in imaging due to renal dysfunction, and the patient's frailty restricted the use of standard immunochemotherapy. Despite partial clinical response to corticosteroids and a single rituximab dose, the patient succumbed to complications of his underlying systemic disease. This case underscores the importance of early recognition of extranodal lymphomas in elderly patients, the challenges in accurate staging and treatment planning in those with poor physiological reserve, and the necessity for individualized, risk-adapted therapeutic strategies. A multidisciplinary approach remains essential to optimize outcomes in such complex and high-risk scenarios.
